# Li_1.2_Mn_0.54_Ni_0.13_Co_0.13_O_2_ nanosheets with porous structure as a high-performance cathode material for lithium-ion batteries†

**DOI:** 10.1039/d1ra06420g

**Published:** 2021-11-15

**Authors:** Zhi Gao, Wenliang Sun, Xiaoliang Pan, Shikun Xie, Lijun Liu, Chengning Xie, Huiling Yuan

**Affiliations:** School of Mechanical Engineering, Jinggangshan University Jian 343009 China xiaoliang_pan@163.com; School of Chemistry and Chemical Engineering, Jinggangshan University Jian 343009 China

## Abstract

The morphological and structural optimizations of electrode materials are efficient ways to enhance their electrochemical performance. Herein, we report a facile co-precipitation and subsequent calcination method to fabricate Li_1.2_Mn_0.54_Ni_0.13_Co_0.13_O_2_ nanosheets consisting of interconnected primary nanoparticles and open holes through the full thickness. By comparing the nanosheets and the agglomerated nanoparticles, the effects of the morphology and structure on the electrochemical performance are investigated. Specifically, the nanosheets exhibit a discharge capacity of 210 mA h g^−1^ at 0.5C with a capacity retention of 85% after 100 cycles. The improved electrochemical performance could be attributed to their morphological and structural improvements, which may facilitate sufficient electrolyte contacts, short diffusion paths and good structural integrity during the charge/discharge process. This work provides a feasible approach to fabricate lithium-rich layered oxide cathode materials with 2D morphology and porous structure, and reveals the relationships between their morphology, structure and electrochemical performance.

## Abstract 2

Li_1.2_Mn_0.54_Ni_0.13_Co_0.13_O_2_ nanosheets with interconnected nanoparticles and open holes are prepared by co-precipitation and calcination processes, exhibiting improved electrochemical performance compared to agglomerated nanoparticles.

## Graphical abstract

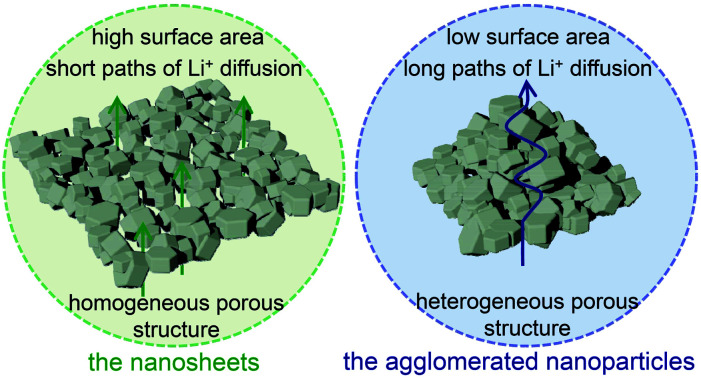

## 1. Introduction

Rechargeable lithium-ion batteries have been widely used in portable consumer electronics, electric vehicles and large-scale energy storage.^1,2^ However, their further development has been limited mainly by the low energy densities of cathode materials.^3^ Thereby, cathode materials with high specific capacities are very appealing for high-energy densities in lithium-ion batteries.

Lithium-rich layered oxide cathode materials with the general formula *x*Li_2_MnO_3_·(1 − *x*)LiMO_2_ (0 < *x* < 1; M = Mn, Ni or/and Co) are regarded as promising candidates for high-energy lithium-ion batteries owing to their large theoretical specific capacity (>250 mA h g^−1^) and high discharge voltage (>3.5 V *vs.* Li/Li^+^).^4,5^ However, lithium-rich layered oxide cathode materials still have great challenges in their large-scale commercial applications, such as relatively poor rate capability, unsatisfactory cycling stability and severe voltage decay on cycling.^6–8^

It has been well acknowledged that the electrochemical properties of lithium-rich layered oxide cathode materials tightly depend on their morphologies and structures.^9–12^ Therefore, numerous efforts have been made to control the morphologies and structures of lithium-rich layered oxide cathode materials for enhancing the rate capability, improving the capacity retention and reducing the voltage decay. For examples, porous Li[Li_0.19_Mn_0.32_Co_0.49_]O_2_ nanorods composed of about 20 nm subunit nanoparticles were synthesized by oxalates co-crystallization and subsequent calcination process, exhibiting the improved charge/discharge performances;^13^ 1D micro- and nanostructured manganese-based bars with differing aspect ratios and compositions were prepared by an ethanol–water mediated co-precipitation method coupled with a post-heat treatment, delivering an excellent reversible capacity and rate capability;^14^ Li_1.2_Mn_0.54_Ni_0.13_Co_0.13_O_2_ nanowires were synthesized *via* a co-precipitation method followed by calcination steps, and achieved a high capacity and excellent capacity retention;^15^ Li_1.140_Mn_0.622_Ni_0.114_Co_0.124_O_2_ microspheres were fabricated by a solvothermal strategy followed by a moderate heat treatment, showing an impressive initial coulombic efficiency and high rate capability;^16^ a hollow porous hierarchical 0.5Li_2_MnO_3_·0.5LiMn_0.4_Co_0.3_Ni_0.3_O_2_ architecture with a delicate flower-like morphology was synthesized by a solvothermal method and subsequent calcination process, and displayed the exceptional electrochemical performance.^17^

These reports demonstrate that lithium-rich layered oxide cathode materials with 1D, 3D and complex hierarchical structures have been commonly fabricated in recent years. But lithium-rich layered oxide cathode materials with 2D hierarchical structure have been rarely reported. Consequently, the syntheses of 2D hierarchical architectures for lithium-rich layered oxide cathode materials still remain a tremendous challenge.

Here, we present a simple route for the fabrication of Li_1.2_Mn_0.54_Ni_0.13_Co_0.13_O_2_ nanosheets with porous structure. The two-dimensional sheet-like architectures own the advantages of open Li^+^ transport path, short diffusion length, high surface area and good structure stability, which are beneficial to large reversible capacity, high rate capability and long-term cycling stability. The relationships among their morphology, structure and electrochemical performance have been further investigated.

## 2. Experimental

### 2.1 Materials syntheses

Porous Li_1.2_Mn_0.54_Ni_0.13_Co_0.13_O_2_ nanosheets were prepared *via* a facile co-precipitation process followed by a calcination treatment. Typically, 8.4 mmol of LiCH_3_COO·2H_2_O, 3.6 mmol of Mn(CH_3_COO)_2_·4H_2_O, 0.87 mmol of Ni(CH_3_COO)_2_·4H_2_O and 0.87 mmol of Co(CH_3_COO)_2_·4H_2_O were dissolved in 30 mL of ethanol, and then 5 mL of NH_3_·H_2_O and 5 mL of diethylene glycol (DEG) were added to the above solution under stirring. Meanwhile, 14.31 mmol of oxalic acid (H_2_C_2_O_4_·2H_2_O) was dissolved in 10 mL of ethanol. Then, the H_2_C_2_O_4_·2H_2_O solution was rapidly poured into the metal acetate mixed solution and kept at room temperature under stirring for 4 h to form MC_2_O_4_·*x*H_2_O (M = Li, Mn, Ni, Co) pink precipitates. The MC_2_O_4_·*x*H_2_O precipitates were dried at 80 °C in air overnight to obtain the precursor. The as-obtained precursor was calcined in air at 450 °C for 5 h and then at 750 °C for 10 h with a heating rate of 3.5 °C min^−1^ to get the Li_1.2_Mn_0.54_Ni_0.13_Co_0.13_O_2_ nanosheets.

For comparison, an independent experiment was conducted to prepare the agglomerated nanoparticles, where ammonium hydrogen carbonate (NH_4_HCO_3_) instead of oxalic acid (H_2_C_2_O_4_·2H_2_O) was used as the precipitant while other conditions were kept identical.

### 2.2 Physical characterizations

The phases of the samples were characterized by X-ray diffraction (XRD) on an X-ray powder diffractometer (Rigaku D/max-rA diffractometer, Cu Kα radiation, *λ* = 1.5406 Å). The XRD refinements were conducted by Rietveld method with FullProf_suite program. The morphologies and structures of the samples were observed by using field-emission scanning electron microscopy (FE-SEM, FEI Quanta 200F) and transmission electron microscopy (TEM, JEOL JEM-2100). The high-resolution TEM (HRTEM) image was obtained on the transmission electron microscopy. The Brunauer–Emmett–Teller (BET) specific surface area and pore size contribution plots were determined by N_2_ adsorption/desorption measurement (Micromeritics ASAP, 2020 M). The chemical compositions of the samples were determined by using inductively coupled plasma-optical emission spectrometry (ICP-OES, Agilent 730).

### 2.3 Electrochemical measurements

The cathode slurry was prepared by using a mixture of the obtained active material, acetylene black and polyvinylidene fluoride (PVDF) with a weight ratio of 80 : 10 : 10 in *N*-methyl-2-pyrrolidone (NMP). After stirring overnight, the slurry was coated uniformly onto an Al foil, dried in an oven at 60 °C, pressed with 2 tons per cm^2^ pressure for 5 min, and punched into circular discs. The circular discs were dried under vacuum at 120 °C for 10 h. The CR2032 coin-type cells were assembled in an argon-filled glovebox by using a metallic lithium foil as the negative electrode, a Celgard 2400 polypropylene as the separator and 1 M LiPF_6_ in a 1 : 1 (volume) mixture of ethylene carbonate (EC) and dimethyl carbonate (DMC) as the electrolyte. Galvanostatic charge/discharge tests were performed at different rates between 2.0 and 4.8 V (1C = 250 mA g^−1^). The measurements of cyclic voltammetry (CV) and electrochemical impedance spectroscopy (EIS) were conducted on a CHI660E electrochemical workstation (Chenhua Instruments Shanghai Inc.). The CV data were recorded with the scan rate of 0.1 mV s^−1^. The EIS data were recorded by applying an ac voltage signal of 5 mV in a frequency range of 0.01–100 000 Hz. All the measurements were carried out at 25 °C.

## 3. Results and discussion

**Fig. 1:**
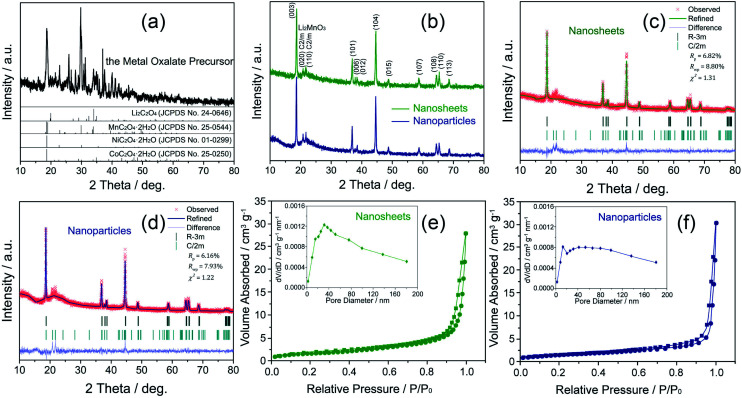
XRD patterns of the metal oxalate precursor obtained by the co-precipitation process (a) and the Li_1.2_Mn_0.54_Ni_0.13_Co_0.13_O_2_ samples after the calcination treatment (b), the Rietveld refinement results (c and d) and the nitrogen adsorption/desorption isotherms ((e and f) the insets are the pore-size distribution curves).

The crystal characteristics of the as-prepared samples were characterized by XRD, SEM and TEM measurements, as shown in Fig. 1. Fig. 1a is the XRD patterns of the metal oxalate precursor obtained by the co-precipitation process. The main diffraction peaks of the metal oxalate precursor can be indexed to Li_2_C_2_O_4_ (JCPDS no. 24-0646), MnC_2_O_4_·2H_2_O (JCPDS no. 25-0544), NiC_2_O_4_·2H_2_O (JCPDS no. 01-0299), and CoC_2_O_4_·2H_2_O (JCPDS no. 25-0250) phases. Fig. 1b shows the XRD patterns of the two samples after the calcination treatment. The main diffraction peaks of the two samples are well indexed to the α-NaFeO_2_-type layered structure with *R*3̄*m* space group (JCPDS no. 09-0063).^18,19^ Some weak reflection peaks around 2*θ* = 20–25° are the characteristic of the superlattice ordering arrangement of Li and Mn ions in the transition metal layers of Li_2_MnO_3_ with *C*/2*m* space group (JCPDS no. 84-1634).^20^ Moreover, the obvious splitting peaks of (006)/(012) and (108)/(110) observed for both samples indicate a good layered structure.^21,22^

To get the detailed information of the structural differences, the XRD patterns of the nanosheets and the nanoparticles are fitted by Rietveld refinement based on *R*3̄*m* structure as shown in Fig. 1c and d. The red crosses display the observed data, while the solid lines are the calculated patterns. The vertical bars present Bragg peak positions of *R*3̄*m* and *C*2/*m*. And the lines at the bottom of the figures show the differences between the observed and calculated patterns. The corresponding lattice constants and the ratios of the peak intensities of (003) and (104) are listed in Table S1.†

Based on the refinement results, there are slight alterations in lattice constants of the two samples. The *c*/*a* values of the two samples are larger than 4.899 (the value of idea cubic close stacking), indicating an enhanced layered structure.^12^ Additionally, the peak intensity ratios of *I*(003)/*I*(104) for the nanosheets and the agglomerated nanoparticles are 1.345 and 1.296, respectively, indicating that both of the samples have a good layered structure and a great cation arrangement between Li^+^ and Ni^2+^.^23^

The nitrogen adsorption–desorption isotherms of the nanosheets and the agglomerated nanoparticles are shown in Fig. 1e and f. The results clearly show that the two samples possess a H1 type absorption–desorption behavior, confirming that the two samples have a porous structure. By comparing the results of the pore-size distribution curves (the insets in Fig. 1e and f), the agglomerated nanoparticles have less pore volume than the nanosheets. Furthermore, the BET specific surface area of the agglomerated nanoparticles (4.967 m^2^ g^−1^) is smaller than that of the nanosheets (6.841 m^2^ g^−1^). From the pore-size distribution curves (the insets in Fig. 1e and f), the size of the major pores in the nanosheets is about 34.6 nm, while the main pore size of the nanoparticles is about 15.3 nm.

The compositions of the two samples were tested by ICP. The Li/Mn/Ni/Co molar ratios are tabulated in Table 1. The compositions of the nanosheets and the agglomerated nanoparticles are Li_1.205_Mn_0.541_Ni_0.131_Co_0.123_O_2_ and Li_1.210_Mn_0.535_Ni_0.130_Co_0.125_O_2_, respectively. The results are approximately consistent with the stoichiometric ratio.

**Table 1:** Li/Mn/Ni/Co molar ratios of the two samples based on ICP-OES analysis

Samples	Li/Mn/Ni/Co molar ratio
Nanosheets	1.205/0.541/0.131/0.123
Nanoparticles	1.210/0.535/0.130/0.125

**Fig. 2:**
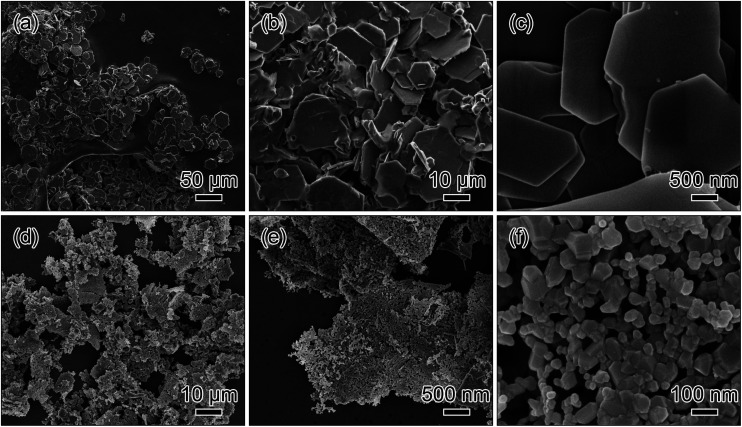
SEM images of the metal oxalate precursor obtained by the co-precipitation process (a–c) and the Li_1.2_Mn_0.54_Ni_0.13_Co_0.13_O_2_ nanosheets after the calcination treatment (d–f).

The morphology and structure of the precursor and the nanosheets were observed by FE-SEM. Fig. 2a–c display that the precursor possesses a sheet-like morphology with the approximate hexagonal shape and the smooth surface. After the calcination treatment, the sheet-like morphology is mainly preserved although some of them are broken into smaller pieces, as shown in Fig. 2d and e. The magnified image in Fig. 2f reveals that the nanosheets with porous structure are consisted of the interconnected primary nanoparticles with an average diameter of ∼100 nm. In bulk system of the precursor, the transition metal oxalates react with lithium oxalate to form the lithium-rich compound, accompanying by the release of some volatile atoms/molecules such as CO and CO_2_. Probably because of the different diffusion rates of these metal elements, the voids form during the calcination reaction. As the reaction proceeds in time, the lithium-rich compound may exhibit the ripening behavior, and the voids may exhibit the merging behaviour, which are driven by the decrease in surface energy. Therefore, the formation of the porous structure in nanosheets can be attributed to synergistic combinations of the Kirkendall effect and the Ostwald ripening which occur during the calcination treatment.^24,25^

**Fig. 3:**
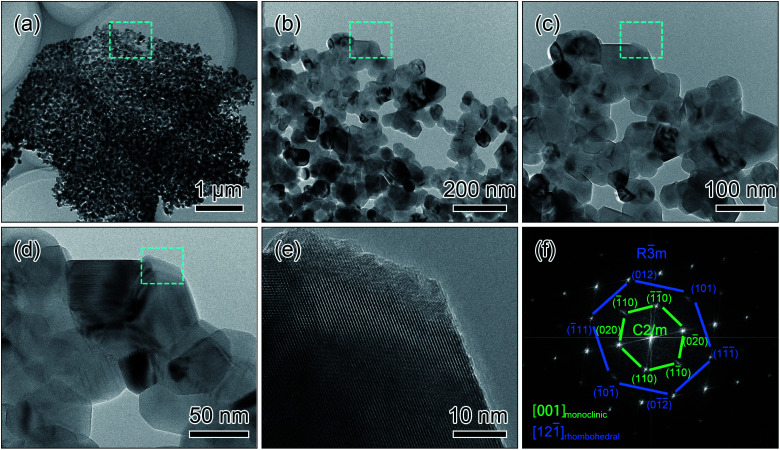
TEM images (a–d), HRTEM image (e) and the corresponding FFT (f) of the nanosheets after the calcination treatment.

The detailed morphology and structure of the nanosheets are observed under TEM and HRTEM. The TEM images in Fig. 3a and b show that the morphology and structure of the nanosheets are in good agreement with the SEM results. The high magnified TEM images in Fig. 3c and d clearly display that there are open holes through the full thickness of the nanosheets. In Fig. 3e, the HRTEM image performed at the edge of the nanoparticle (the dashed region in Fig. 3d) displays the noticeable lattice fringes, and the diffraction information is extracted by the Fast Fourier Transformation (FFT) technique. The corresponding FFT pattern in Fig. 3f can be indexed to the diffractions along equivalent zone axes of [121̄] direction in rhombohedral structure (space group *R*3̄*m*) and [001] direction in monoclinic structure (space group *C*2/*m*), which has been proved by many literatures.^9,11,26^ The structural analyses of the HRTEM image and the FFT pattern combined with the results of XRD pattern suggest that the *R*3̄*m* and *C*2/*m* phases are co-existed in the nanosheets.

**Fig. 4:**
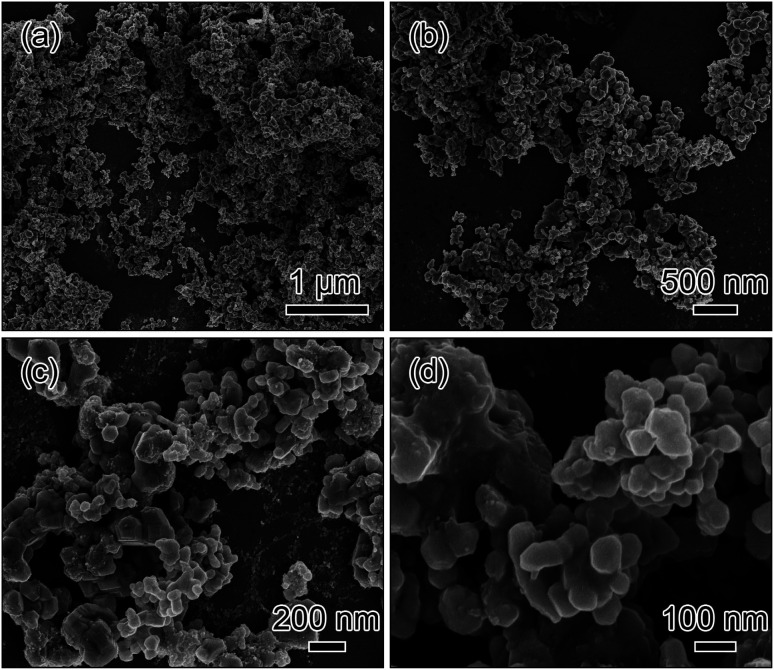
SEM images of the agglomerated nanoparticles fabricated by NH_4_HCO_3_ instead of H_2_C_2_O_4_·2H_2_O.

For comparison, the agglomerated nanoparticles were fabricated, where 14.31 mmol NH_4_HCO_3_ instead of 14.31 mmol H_2_C_2_O_4_·2H_2_O was used as the precipitant while other conditions were kept identical as shown in Fig. 4. From the low magnification SEM images in Fig. 4a and b, it is observed that the sample is made up of the randomly agglomerated particles. From the highly magnified SEM images in Fig. 4c and d, it is obvious that the randomly agglomerated particles have an average diameter of about 100 nm.

**Fig. 5:**
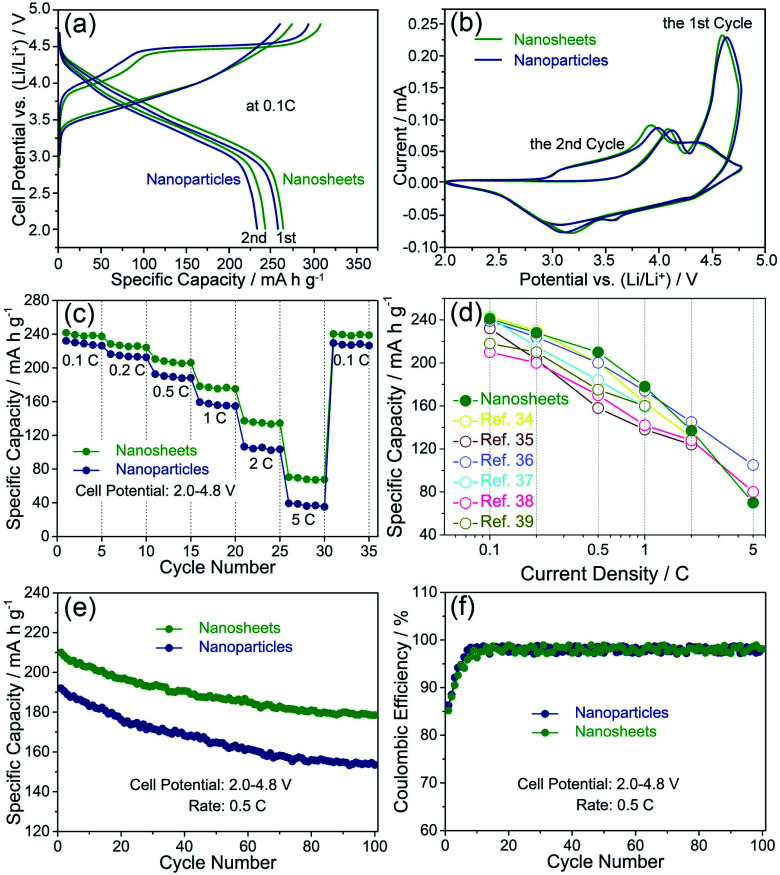
Electrochemical performance of the nanosheets and the agglomerated nanoparticles performed in the voltage range of 2.0–4.8 V *vs.* Li/Li^+^: the charge/discharge curves at 0.1C (a), CV plots at a scan rate of 0.1 mV s^−1^ (b), rate capability at various rates (c), the comparison of the discharge capacities (d), cycling properties (e) and coulombic efficiency at 0.5C (f).

To demonstrate the morphological and structural advantages of the nanosheets, the as-prepared samples were investigated as cathode materials for lithium-ion batteries as shown in Fig. 5.

Fig. 5a displays the initial and second charge/discharge profiles of the nanosheets and the agglomerated nanoparticles at a rate of 0.1C in the voltage range of 2.0–4.8 V. Both of the samples reveal similar initial charge and discharge profiles. During the first charge process, two typical voltage plateaus can be observed. The sloping voltage plateau below 4.5 V is associated to the reversible delithiation process combined with the oxidations of Ni^2+^/Ni^3+^/Ni^4+^ and Co^3+^/Co^4+^, while the long voltage plateau above 4.5 V is related to the irreversible oxygen loss together with Li^+^ extraction from the activation of Li_2_MnO_3_ region.^27,28^ Specially, the initial discharge capacity of the nanosheets reaches 265 mA h g^−1^, while the agglomerated nanoparticles deliver the discharge capacity of 258 mA h g^−1^. Both of the samples show a discharge capacity loss during the second cycling. The discharge capacities of the nanosheets and the agglomerated nanoparticles are 241 and 232 mA h g^−1^, respectively.

To further investigate the detailed information about the changes in the initial and second charge/discharge profiles of the two samples, cyclic voltammograms (CV) tests were conducted at a scan rate of 0.1 mV s^−1^ with a voltage range from 2.0 to 4.8 V as shown in Fig. 5b. For the initial charge process, both of the samples have two main oxidation peaks at about 4.1 V and 4.6 V. The oxidation peak at low potential is attributed to the delithiation process, and the oxidation peak at high potential is ascribed to the activation of Li_2_MnO_3_ region which is an irreversible electrochemical reaction.^29,30^ For the first discharge process, there are a weak peak at about 4.4 V and two broad peaks at about 3.6 V and 3.2 V. These reduction peaks are related to the reduction reactions of Ni^2+^/Ni^3+^/Ni^4+^, Co^4+^/Co^3+^ and Mn^4+^/Mn^3+^.^31,32^ For the following cycle, it can be found that the oxidation peaks at 4.6 V almost disappear, which fully indicates the irreversible oxygen loss.^33^ By the comparisons of the positions and intensities of the oxidation and reduction peaks, the nanosheets exhibit smaller voltage differences and higher intensity values than those of the agglomerated nanoparticles, indicating that the nanosheets possess a less polarization during the charge/discharge process.

The rate capabilities of the nanosheets and the agglomerated nanoparticles were performed at different rates ranging from 0.1C to 5C between 2.0 and 4.8 V as shown in Fig. 5c. The discharge capacities of the two samples exhibit the same trend of decreasing with the increasing rates. In detail, the typical discharge capacities of the nanosheets can achieve 241 mA h g^−1^ at 0.1C, 228 mA h g^−1^ at 0.2C, 210 mA h g^−1^ at 0.5C, 178 mA h g^−1^ at 1C, 137 mA h g^−1^ at 2C and 70 mA h g^−1^ at 5C. When the rate reverts to 0.1C, the discharge capacity can still remain 240 mA h g^−1^. In comparison, the agglomerated nanoparticles exhibit the inferior rate capabilities, delivering the discharge capacities of 232, 216, 192, 159, 106 and 39 mA h g^−1^ at 0.1, 0.2, 0.5, 1 and 5C, respectively. And only 229 mA h g^−1^ is recovered back to 0.1C. Furthermore, the comparisons between the discharge capacities of Li_1.2_Mn_0.54_Ni_0.13_Co_0.13_O_2_ cathodes reported by other groups and our work are shown in Fig. 5d. It can be seen that the nanosheets in this work deliver the comparable discharge capacities with those reported in the ref. 34–39. It demonstrates that the 2D morphology and porous structure of the nanosheets facilitate the achievement of their high discharge capacities in lithium-rich layered oxide cathodes.

The cycling stabilities of the nanosheets and the agglomerated nanoparticles were conducted for 100 cycles at 0.5C in the voltage range of 2.0–4.8 V as shown in Fig. 5e. After 100 cycles, the nanosheets show a reversible capacity of 179 mA h g^−1^ with a capacity retention of 85%, while the agglomerated nanoparticles only have a reversible capacity of 154 mA h g^−1^ with 20% capacity loss. The results clearly show that the nanosheets own a better cycling stability than the agglomerated nanoparticles. In addition, coulombic efficiency refers to the ratio (as a percentage) of the battery discharge capacity to the charge capacity during the same cycle.^40^ The coulombic efficiency of the nanosheets and the nanoparticles is shown in Fig. 5f. During the initial few cycles, a low coulombic efficiency of the two samples can be observed, which is closely related with the irreversible electrochemical activation of Li_2_MnO_3_. In the subsequent cycles, their coulombic efficiency becomes stable, reaching approximately 98%. More studies will be done to determine the exact processes in the future.

**Fig. 6:**
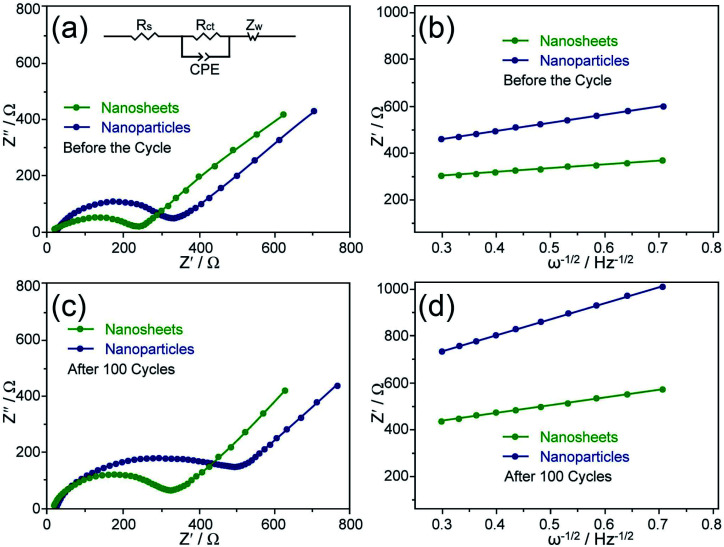
Nyquist plots obtained before the cycle (a) and after 100 cycles (c) at 0.5C with the full discharging state (the inset is the equivalent circuit), and the relationship plots between *Z*′ and *ω*^−1/2^ at low frequency region before the cycle (b) and after 100 cycles (d) for the nanosheets and the agglomerated nanoparticles.

To further verify the improved reaction kinetics of the nanosheets, electrochemical impedance spectra (EIS) was conducted before the cycle and after 100 cycles at 0.5C with the full discharging state. Nyquist plots of the two samples can be observed in Fig. 6. The inset in Fig. 6a shows the corresponding equivalent circuit for fitting. Generally, the intercept in the high-frequency range represents the solution resistance (*R*_s_); the quasi-semicircle corresponds to the charge transfer resistance (*R*_ct_); the constant phase element is relate to the double layer capacitance (CPE); and the sloping line is ascribed to Warburg impedance (*Z*_w_).^41,42^ Based on the observation in Fig. 6a, the nanosheets have a smaller semicircle and a more vertical straight line than the agglomerated nanoparticles, indicating a lower charge transfer resistance and a faster Li^+^ diffusion behavior. In detail, *R*_s_ and *R*_ct_ of the nanosheets are 18.1 and 240.7 Ω according to the results of the equivalent circuit fitting, respectively, while the agglomerated nanoparticles are 27.3 and 329.2 Ω. According to the slopes (*σ*) in Fig. 6b, the values of the nanosheets and the agglomerated nanoparticles are 153 and 342 Ω cm^2^ s^−1/2^, respectively. Based on the Warburg equations,^28,31,43^ Li^+^ diffusion coefficients of 2.55 × 10^−16^ and 5.11 × 10^−17^ cm^2^ s^−1^ can be calculated for the nanosheets and the agglomerated nanoparticles, respectively. After 100 cycles, Li^+^ diffusion coefficients of the nanosheets and the agglomerated nanoparticles are 5.80 × 10^−17^ and 1.24 × 10^−17^ cm^2^ s^−1^, respectively. The EIS results reveal that the nanosheets have a high coefficient and a small change in Li^+^ diffusion behavior, which indicate an easy diffusion accessibility and a good structural stability during the cycles.

**Fig. 7:**
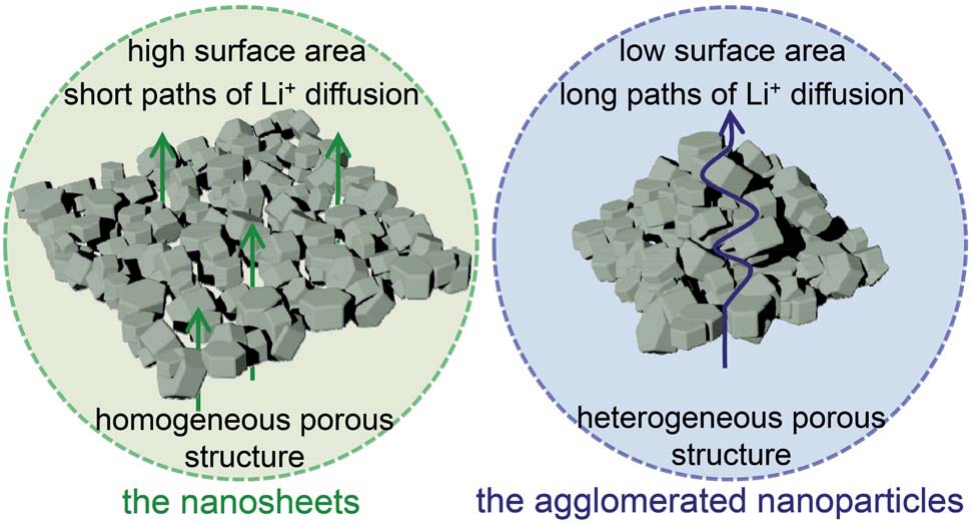
Schematic illustration of the advantages of the nanosheets compared with the agglomerated nanoparticles.

Based on the above results of electrochemical measurements, it can be concluded that the nanosheets display the improved electrochemical properties with large specific capacity, high rate capability and good cycle life. The fundamental cause of this improved electrochemical performance could be ascribed to their merits of the morphology and structure. For the nanosheets, their sheet-like morphology possesses a high specific surface area, which enable the sufficient contact between electrode and electrolyte. And their open pores through the full thickness can effectively shorten the Li^+^ diffusion paths from the surface to the center region of the nanosheets. Furthermore, the sufficient electrolyte contact and short diffusion paths can greatly facilitate Li^+^ transport kinetics and the reduction of electrode polarization resistance, resulting in the enhancement of specific capacity and rate performance. Meanwhile, their homogeneous porous structure with uniform arrangement of the interconnected primary nanoparticles can relieve the volume change during Li^+^ intercalation/deintercalation through coordinating expansion/contraction, and confirm the good structural integrity of the geometrical configuration, leading to the improvement of cycling stability. In comparison, the agglomerated nanoparticles have a low specific surface area, possess long diffusion distances from the surface to the center region, and show a heterogeneous porous structure, which limit their reaction kinetics and cycling stability. Consequently, the advantages of their homogeneous porous structure and sheet-like morphology coupled with high surface area and open holes for the nanosheets could afford the overall achievements of their superior electrochemical properties, which are illustrated in Fig. 7.

## 4. Conclusions

In summary, we report a facile strategy to fabricate Li_1.2_Mn_0.54_Ni_0.13_Co_0.13_O_2_ nanosheets with porous structure by a co-precipitation process followed by a calcination treatment. Electrochemical measurements indicate that the nanosheets achieve the improved electrochemical properties in comparison to the agglomerated nanoparticles. Particularly, the nanosheets exhibit a discharge capacity of 210 mA h g^−1^ at 0.5C with a capacity retention of 85% after 100 cycles. The enhanced electrochemical performance of the nanosheets could be contributed to their morphological and structural advantages, providing sufficient electrolyte contacts, short diffusion paths and good structural integrity. This work provides a feasible strategy for the design and preparation of lithium-rich layered oxides with the 2D morphology and porous structure using in lithium-ion batteries.

## Conflicts of interest

There are no conflicts to declare.

## Supplementary Material

RA-011-D1RA06420G-s001
