# Supplementary material for: Li1.2Mn0.54Ni0.13Co0.13O2 nanosheets with porous structure as a high-performance cathode material for lithium-ion batteries
Source: RSC Adv. 2021 Nov 15;11(58):36588–95. doi: 10.1039/d1ra06420g (PMC9043626; doi:10.1039/d1ra06420g)
Supplement: RA-011-D1RA06420G-s001 [file RA-011-D1RA06420G-s001.pdf]

Electronic Supplementary Material (ESI) for RSC Advances.
This journal is © The Royal Society of Chemistry 2021

Supplementary Information

$Li_{1.2}Mn_{0.54}Ni_{0.13}Co_{0.13}O_2$ nanosheets with porous structure as high-performance cathode material for lithium-ion batteries

Zhi Gao,[a] Wenliang Sun,[a] Xiaoliang Pan, *[a] Shikun Xie,[a] Lijun Liu,[b] Chengning Xie,[a] and Huiling Yuan[a]

a. *School of Mechanical Engineering, Jinggangshan University, Jian, 343009, China.*

b. *School of Chemistry and Chemical Engineering, Jinggangshan University, Jian, 343009, China.*

*Corresponding author:

E-mail address: xiaoliang_pan@163.com (X. L. Pan)

Table S1 The Rietveld refinement data obtained from XRD.

Samples	Lattice Constants							R_p/%	R_{wp}/%	χ^2	$I_{(003)}/I_{(104)}$
	a/Å	c/Å	c/a ratio	V/ Å^3	α	β	γ				
Nanosheets	2.852	14.166	4.967	99.785	90°	90°	120°	6.82	8.80	1.31	1.345
Nanoparticles	2.855	14.135	4.951	99.776	90°	90°	120°	6.16	7.93	1.22	1.296
